# Relative Roles of Grey Squirrels, Supplementary Feeding, and Habitat in Shaping Urban Bird Assemblages

**DOI:** 10.1371/journal.pone.0109397

**Published:** 2014-10-22

**Authors:** Colin Bonnington, Kevin J. Gaston, Karl L. Evans

**Affiliations:** 1 Department of Animal and Plant Sciences, University of Sheffield, Sheffield, United Kingdom; 2 Environment and Sustainability Institute, University of Exeter, Penryn, Cornwall, United Kingdom; University of Lleida, Spain

## Abstract

Non-native species are frequently considered to influence urban assemblages. The grey squirrel *Sciurus carolinensis* is one such species that is widespread in the UK and is starting to spread across Europe; it predates birds’ nests and can compete with birds for supplementary food. Using distance sampling across the urbanisation intensity gradient in Sheffield (UK) we test whether urban grey squirrels influence avian species richness and density through nest predation and competition for supplementary food sources. We also assess how urban bird assemblages respond to supplementary feeding. We find that grey squirrels slightly reduced the abundance of breeding bird species most sensitive to squirrel nest predation by reducing the beneficial impact of woodland cover. There was no evidence that grey squirrel presence altered relationships between supplementary feeding and avian assemblage structure. This may be because, somewhat surprisingly, supplementary feeding was not associated with the richness or density of wintering bird assemblages. These associations were positive during the summer, supporting advocacy to feed birds during the breeding season and not just winter, but explanatory capacity was limited. The amount of green space and its quality, assessed as canopy cover, had a stronger influence on avian species richness and population size than the presence of grey squirrels and supplementary feeding stations. Urban bird populations are thus more likely to benefit from investment in improving the availability of high quality habitats than controlling squirrel populations or increased investment in supplementary feeding.

## Introduction

Urbanisation is one of the fastest growing land uses, and generates environments with very different selection pressures than the rural ones which it replaces [1]–[3]. The resultant species assemblages in towns and cities thus also differ markedly in their structure and composition than equivalent assemblages in more rural environments [4]–[6], but can include considerable populations of some species of conservation concern [7]. Numerous factors can drive this divergence in assemblage structure, with changes in habitat quality and the introduction of non-native species frequently considered important [8]–[9]. Much of the research on urban assemblage structure has focused on avian assemblages, which can also be influenced by predation risk and provision of supplementary food [10]–[12].

In the UK, the grey squirrel *Sciurus carolinensis* is an exotic species that is widespread in urban areas [13]. Grey squirrels sometimes predate birds’ nests [14], and there is evidence that they can out-compete birds for supplementary food [15], [16]. As a consequence of these observations concern has been expressed that grey squirrels may limit avian population size, but empirical evidence is very limited. In rural areas the populations of a small number of bird species are slightly reduced when grey squirrels are present with the assumption being that this is due to nest predation [17]–[18], but empirical data are lacking from urban areas.

The provision of supplementary food for wild birds is a widespread activity in many developed countries, with almost half of UK households providing food for birds [19]. This activity is associated with grey squirrel occurrence [13]. Positive associations have also been documented between supplementary feeding and the size of wintering and breeding bird populations [10], [11]. Such positive associations may arise through improved survival rates or increased productivity [20], [21]. Some recent studies have, however, found that supplementary feeding is associated with reduced reproductive success [22], and that provision of low quality food can reduce maternal investment in egg quality [23]. Moreover, there is much spatial variation in the effects of supplementary feeding on avian population size, with positive effects in northern Europe (Finland, UK) but negligible effects in central Europe (France), and not all species that use feeders exhibit increased population size [10]–[11]; [24]–[25]. One possible mechanism for this is that supplementary feeding promotes interference competition with a small number of dominant aggressive species monopolising resources which could reduce the benefits they provide to native species [26], [27]. Indeed, experimental evidence demonstrates that grey squirrel presence at supplementary feeding stations severely limits food intake rates (lowered by over 90%) of a wide range of passerines demonstrating the potential for strong interference competition [16]. It is unknown, however, if such competition influences avian population size, for example this would not be the case if feeding stations are sufficiently abundant that displaced birds can readily find alternative stations that lack squirrels or if the availability of such stations does not regulate bird population size.

Our primary objective is to assess if grey squirrels influence the structure of urban avian assemblages through either nest predation or competition at supplementary feeding stations. We do so by comparing breeding and wintering avian assemblage structure across multiple urban locations, in which grey squirrels are both absent and present, whilst taking the amount of green space, its quality, and provision of supplementary food into account. In so doing we also provide an additional assessment of the association between supplementary feeding and avian assemblage structure. We use Sheffield (UK), the fifth largest urban municipality in the UK, as a case study.

## Materials and Methods

No specific permissions were required to conduct this work. Research did not involve endangered or protected species or collection of biological material.

### Sampling approach

This study was conducted in urban Sheffield, which contains c. 555,500 people [28]. Urban areas were defined as 1 km×1 km squares with at least 25% coverage of hard surface. This definition has been used in numerous other urban ecology studies conducted in the Sheffield region, and is appropriate as it excludes areas of the countryside which fall within the city’s administrative boundaries, but retains all parks and other green-spaces surrounded by built up land within the urban landscape. This resulted in 143 1 km×1 km squares, each of which was split into 16 cells of 250 m×250 m. The green space cover (%) of each of these smaller cells was determined using OS Mastermap digital maps (EDINA Digimap, Edinburgh, UK) in ArcMap 9.3 (ESRI Corporation, Redlands, CA, USA). Each cell was assigned to one of ten categories, based on its green space cover (%) ranging from category 1 (0–10% green space) to category 10 (91–100% green space). Ten sampling points were then selected using a random stratified approach, in each category of green space, resulting in 100 sampling points. An additional 40 sampling points were selected from cells with intermediate amounts of green space (41–80% green space) to increase sampling effort in those areas most likely to have variation in squirrel occurrence.

### Field observations

Visual surveys and counting dreys in the winter are the two most economical methods for surveying squirrels [29]. Drey counts require data on the mean number of dreys used by an individual squirrel, but such data are unavailable for urban areas. Moreover, in towns and cities grey squirrel dreys can be very difficult to detect as they are frequently built inside old corvid nests, the roof space of buildings and evergreen trees [30], which reduces the method’s value. We thus assess grey squirrel distributions using a visual survey method. We used a fixed duration point count methodology which has previously been used to estimate densities of other squirrel species [31]–[33]. We followed reference [32] and used a ten minute point count; we used a fixed radius of 100 m [34]. The size of the point count survey radius is thus approximately 3 ha, which incorporates the typical home range size (0.5 to 3 hectares) of grey squirrels occupying patches of fragmented habitat [35]. Our methodology is described in full in [13]. These point counts were also used to sample the avian assemblage; such methods have previously been frequently used to sample urban avian assemblages in both the breeding and non-breeding season [36], [37].

Each sampling point was visited four times in 2010 on dry calm days (less than 4 on the Beaufort scale), once every season (winter: 4 February –2 March; spring: 8–27 April; summer: 5–21 July; autumn: 25 October –10 November). Surveys were not conducted during very cold days (<3°C) as these can reduce squirrel activity. Sampling points were located in the field using a handheld GPS receiver and a map of each location from Google Earth. The exact sampling point was accessible in 102 out of 140 cases (73%); when it was not accessible, the observer (C. Bonnington) stood at the nearest accessible point within the same cell. Ten-minute avian point counts of a fixed radius of 100 m were conducted in daylight hours within 5 hours of sunrise or 5 hours of sunset, during each season. Observations began immediately on arrival at the point location. For each detected individual, the species, radial distance from the observer (within the 100 m point count radius) and detection type (whether seen or heard) were recorded. Distances were recorded using a range finder (Bushnell Laser range finder Sport 450, Overland Park, KS, USA). Grey squirrels and actively stocked supplementary feeding stations were recorded in the same manner as the avifauna, but if no squirrel or feeding station was recorded during the point count period the survey area was searched for a maximum of 10 minutes to confirm the absence of squirrels and actively stocked feeding stations.

The habitat characteristics of each survey area were recorded: the height of the 20 closest trees (>2 m high and ≥20 cm diameter at breast height in survey area) to the sampling point; canopy cover (%) of the survey area estimated from aerial Google Earth maps (using imagery recorded in summer 2008); and green space (%) of the survey area from OS Mastermap (see above). Ground-truthing during the surveys confirmed the estimates of canopy cover and green space.

### Density estimates

Distance software (v.6, [38]) was used to calculate avian densities (number/hectare) at each sampling point. We excluded a small number of rarely detected species that exclusively use habitats that are not used by grey squirrels: wetlands (mallard *Anas platyrhynchos*, 2 detections; coot *Fulica atra*, 1 detection; moorhen *Gallinula chloropus*, 4 detections; sand martin *Riparia riparia*, 20 detections; sedge warbler *Acrocephalus schoenobaenus*, 2 detections) and open fields (skylark *Alauda arvensis*, 1 detection). Following standard distance sampling protocols [34] we fitted half-normal and hazard-rate distributions to all bird datasets to model how detection declined with distance from bird observations, across the four seasons. Urban form (green space % of the survey area), detection type (visual or audial) and season were included as covariates in models of detectability functions. The grouping of appropriate distance bands was explored until a good fit between each model and the data was obtained, by comparing the modelled detection function against the observed distance data, the goodness-of-fit statistics and Akaike’s Information Criterion values for alternative grouping (and covariate combinations) and detection function options. For those species with fewer than 32 observations detectability functions were constructed by including observations of a surrogate species following reference [39]. The best fitting models were used to generate density estimates for each avian species, at all sampling points, in each season (Table S1). The same approach was used to calculate densities of grey squirrels and actively stocked supplementary feeding stations. There was, however, limited variation between point counts in the resultant density estimates so subsequent statistical analyses were restricted to using the presence/absence of grey squirrels and feeding stations as predictors (respectively termed squirrel occurrence and supplementary feeding stations).

### Species classifications

Species were classified as using supplementary food if they were recorded as doing so in at least 75% of gardens in the BTO’s Garden Bird Feeding Survey (GBFS) in the last four years (http://www.bto.org/volunteer-surveys/gbfs/results). Data are not available from this survey for a small number of our recorded species (feral pigeon *Columba livia*, carrion crow *Corvus corone* and most summer migrants). The feeder use of these species was classified following Fuller *et al.* (2008). The resultant classification (Table S1) is identical to reference [11] except that we also classify coal tit *Periparus ater* and long-tailed tit *Aegithalos caudatus* as using supplementary feeders, these species take supplementary food in respectively 90% and 75% of gardens in the GBFS. Species that were present during the winter were classified in two categories of sensitivity to interference competition, i.e. most and least sensitive (Table S1). The most sensitive species comprised those that met all of the following criteria: i) they used supplementary feeders (as defined above); ii) obtained most of their supplementary food from raised or hanging feeding stations, rather than feeding on the ground, as these are the types of feeders typically used by grey squirrels; and iii) were unaggressive small bodied species. These criteria are derived from the results of a food competition experiment which demonstrated that small bodied avian passerines, with the exception of the aggressive robin *Erithacus rubecula*), that used hanging feeders were susceptible to interference competition from grey squirrels [16]. All other species were classified as least sensitive to food competition from grey squirrels.

Species detected during the breeding season were classified as most or least sensitive to grey squirrel predation (Table S1). Species were classified as most sensitive if they nested in habitats frequently used by grey squirrels, and their daily nest failure rates were greater than 1% nest/day as calculated from the British Trust for Ornithology’s nest record card scheme [40] as the vast majority of nest failures are caused by predation in our focal species. Data for pheasant *Phasianus colchicus* were obtained from the Game and Wildlife Conservation Trust [41]. Using this methodology cavity nesters and aggressive large bodied species were typically classified as least sensitive to predation, whilst other species were classified as being most sensitive to predation. There was a clear gap in the distributions of daily nest predation rates between the two groups of species (most sensitive species: 1.1 to 4.27%; least sensitive species: 0.08 to 0.74%).

### Statistical analysis

Analyses were conducted to assess how grey squirrels and supplementary feeding influenced the structure of urban avian assemblages during the breeding and wintering seasons. Although surveys were conducted in all four seasons, we focus on assessing how the breeding and wintering avifaunas were influenced by grey squirrels as these are likely to be most susceptible respectively to nest predation and food competition by grey squirrels. Data on the composition of the avifauna during the breeding season used observations from the spring point counts for the vast majority of species, but for a small number of late arriving summer migrants (Table S1) the summer survey data were used as some individuals would not have arrived at their breeding sites by the time of the spring survey period.

R v. 2.15.1 [42] was used for all statistical analyses. The species richness and density of each avian assemblage (i.e. species grouped by their sensitivity categories) was modelled, using generalised linear models, as a function of squirrel occurrence, mean tree height (m), canopy cover (%), green space in the 250 m×250 m cell (%), and presence of supplementary feeding stations. Interaction terms between squirrel occurrence and other predictors were included to assess whether squirrels altered the slope of the relationships between assemblage structure (i.e. species richness and density) and key predictor variables, i.e. presence of supplementary feeding stations, and measures of habitat quality. This is important because positive relationships between predators and their avian prey have been reported by other studies [12], [17], [18], probably reflecting a mutual preference for similar habitats, which may mask any suppression of population densities by grey squirrels.

The above analyses provide some information on the influence of supplementary feeding stations on assemblage structure, but to assess this further additional analyses were conducted that modelled the species richness and density of species that use and do not use supplementary feeders as a function of the presence of these feeders, mean tree height, canopy cover and green space. These analyses build on previous analyses of this issue by using empirical data on active feeder occurrence at the focal survey site and considering a wider range of habitat indicators than only the amount of green space.

Preliminary exploration indicated that all relationships with continuous predictors were linear and thus square terms were not included as predictors. Tolerance values of all predictors were sufficiently above the threshold (0.1; minimum 0.48 for canopy cover) below which correlations between predictors can bias the results of multiple regressions [43]. Model selection for all generalised linear models adopted an information theoretic approach; we constructed all possible models given the suite of our predictor variables using the MuMIn package, and the 95% confidence set of models comprised those whose cumulative weights summed to 0.95. Model averaging was conducted across this set of models to assess the influence of all predictors on avian species richness and density for each category of species. We used the spdep package to test all response variables for spatial autocorrelation following the methodology of Dormann *et al.* [44]. Moran’s I tests demonstrated that spatial autocorrelation was extremely limited (maximum Moran’s I = 0.037, for the breeding species richness of species most sensitive to nest predation), and non-significant for the majority of response variables. For those response variables with statistically significant Moran’s I values comparison of full models constructed with and without taking spatial autocorrelation into account indicated that spatial autocorrelation had limited influence on parameter estimates and explanatory power (Tables S2 & Tables S3). We thus only report the results from non-spatial models.

## Results

### Breeding season - sensitivity to nest predation

Multiple regression models explained between one sixth and just over a third of the variation in the richness and density, respectively, of breeding avian assemblages (Table 1). There was no evidence that the presence of grey squirrels, as a main effect, was negatively associated with avian species richness or density; indeed, squirrel occurrence was positively associated with the richness and density of those bird species most sensitive to grey squirrel nest predation, but explanatory capacity was limited (respective model averaged partial r^2^ = 0.06 and 0.07; Table 1). The richness and density of these species was negatively influenced by the interaction between squirrel occurrence and canopy cover, indicating that grey squirrel presence reduced the benefits of increasing woodland cover for these species, but explanatory capacity was again limited (respective model averaged partial r^2^ = 0.05 and 0.04; Table 1). For the richness and density of those least sensitive bird species, interaction terms between squirrel occurrence and other predictors had negligible explanatory capacity (model averaged partial r^2^<0.02; Table 1).

**Table 1:** Generalised linear models of relationships between avian species richness and density and grey squirrel occurrence during the breeding season, whilst taking habitat and occurrence of supplementary feeders into account.

		Canopy cover		Mean tree height		Supplementary feedingstations		Green space		Squirreloccurrence		Interactions with squirreloccurrence	
Avian sensitivity categoryresponse variable	ModelR^2^	PartialR^2^	Mean ± s.e.	PartialR^2^	Mean ± s.e.	PartialR^2^	Mean ± s.e.	PartialR^2^	Mean ± s.e.	PartialR^2^	Mean ± s.e.	PartialR^2^	Mean ± s.e.
Most sensitive species richness	0.352	0.115	0.056±0.012	0.008	−0.046±0.057	0.001	−0.098±0.212	0.042	0.021±0.008	0.064	3.343±1.172	0.049	−0.063±0.022^d^
Most sensitive density	0.326	0.097	0.135±0.035	0.001	−0.037±0.083	0.007	0.654±0.950	0.023	0.032±0.027	0.068	11.587±4.544	0.042	−0.090±0.078^d^
Least sensitive species richness	0.163	0.005	0.004±0.006	0.003	−0.012±0.023	0.064	0.705±0.229	0.040	0.011±0.005	0.005	0.448±0.695	0.003	−0.004±0.007^c^
Least sensitive density	0.162	0.002	−0.010±0.024	0.005	−0.101±0.167	0.102	5.405±1.696	0.021	0.044±0.036	0.021	0.126±3.499	0.014	−3.352±4.070^a^

Species are classified by their relative sensitivity to grey squirrel nest predation. An information theoretic approach to model selection was adopted and data reported are model averaged values; standard errors are unconditional. In the final column, the partial R^2^ is for all relevant interactions combined, and the mean ± s.e. are the model averaged values for the interaction with the highest explanatory power (and thus contributing the most to the combined partial R^2^ in this column). Superscript letters are given to represent which interaction term has the highest explanatory power: ^a^ supplementary feeding stations*squirrel occurrence; ^b^ mean tree height*squirrel occurrence; ^c^ green space* squirrel occurrence, ^d^ canopy cover*squirrel occurrence.

Predictors other than grey squirrel occurrence, and its interactions, typically explained more of the variation in the response variables for both the most and least sensitive species to grey squirrel nest predation. Canopy cover was the habitat predictor that explained most of the variation in species richness and density (respective model averaged partial r^2^ = 0.12 and 0.1; Table 1) of species most sensitive to grey squirrel nest predation. The presence of supplementary feeding stations explained the most variation in species richness and density (respective model averaged partial r^2^ = 0.06 and 0.1; Table 1) of species least sensitive to grey squirrel nest predation. The proportion of green space influenced the richness and density of the most and least sensitive bird species to nest predation, although the explanatory capacity was limited (model averaged partial r^2^<0.05; Table 1).

### Winter - food competition

Multiple regression models explained between one sixth and a third of the variation in the richness and density, respectively, of wintering avian assemblages (Table 2). There was no evidence that the presence of grey squirrels, as a main effect, was negatively associated with avian species richness or density; indeed, squirrel occurrence was positively associated with the richness of those bird species most sensitive to food competition with grey squirrels, but explanatory capacity was limited (model averaged partial r^2^ = 0.03; Table 2). There was very limited evidence that the interaction between squirrel occurrence and green space influenced the richness of the most sensitive bird species (model averaged partial r^2^ = 0.02; Table 2). For the density of those most sensitive bird species, and the richness and density of those bird species least sensitive to food competition, interaction terms between squirrel occurrence and other predictors had negligible explanatory capacity (model averaged partial r^2^<0.01; Table 2).

**Table 2:** Generalised linear models of relationships between avian species richness and density and grey squirrel occurrence during the winter, whilst taking habitat and occurrence of supplementary feeders into account.

		Canopy cover		Mean tree height		Supplementary feedingstations		Green space		Squirreloccurrence		Interactions with squirreloccurrence	
Avian sensitivity categoryresponse variable	ModelR^2^	PartialR^2^	Mean ± s.e.	PartialR^2^	Mean ± s.e.	PartialR^2^	Mean ± s.e.	PartialR^2^	Mean ± s.e.	PartialR^2^	Mean ± s.e.	PartialR^2^	Mean ± s.e.
Most sensitive species richness	0.306	0.137	0.032±0.008	0.017	−0.051±0.040	0.018	−0.229±0.255	0.027	0.010±0.006	0.025	1.462±1.363	0.017	−0.016±0.021^c^
Most sensitive density	0.285	0.161	0.196±0.043	0.003	−0.097±0.158	0.004	−0.803±1.225	0.016	0.033±0.034	0.007	3.307±4.768	0.002	−0.029±0.055^c^
Least sensitive species richness	0.197	0.005	0.006±0.010	0.018	0.079±0.070	0.039	1.064±0.462	0.052	0.024±0.009	0.012	2.216±2.472	0.009	−0.018±0.028^c^
Least sensitive density	0.129	0.001	−0.001±0.021	0.002	0.088±0.185	0.072	7.922±2.490	0.026	0.088±0.059	0.004	4.483±8.101	0.004	−1.863±3.246^a^

Species are classified by their relative sensitivity to food competition from grey squirrel. An information theoretic approach to model selection was adopted and data reported are model averaged values; standard errors are unconditional. In the final column, the partial R^2^ is for all relevant interactions combined, and the mean ± s.e. are the model averaged values for the interaction with the highest explanatory power (and thus contributing the most to the combined partial R^2^ in this column). Superscript letters are given to represent which interaction term has the highest explanatory power: ^a^ supplementary feeding stations*squirrel occurrence; ^b^ mean tree height*squirrel occurrence; ^c^ green space* squirrel occurrence, ^d^ canopy cover*squirrel occurrence.

Predictors other than grey squirrel occurrence, and its interactions, typically explained more of the variation in the response variables for both the most and least sensitive species to food competition with grey squirrels. Canopy cover was the habitat predictor that explained most of the variation in species richness and density (respective model averaged partial r^2^ = 0.14 and 0.16; Table 2) of species most sensitive to food competition. The proportion of green space and the presence of supplementary feeding stations explained most of the respective variation in species richness and density (respective model averaged partial r^2^ = 0.05 and 0.07; Table 2) of species least sensitive to food competition.

### Supplementary feeding effects during the breeding season and winter

When taking habitat factors into account the presence of supplementary feeding stations had very limited influence on the species richness (model averaged partial r^2^ = 0.03) and density (model averaged partial r^2^ = 0.04) of wintering avian assemblages of species that regularly use supplementary feeders (Table 3). We found no evidence to suggest that supplementary feeding stations explained any of the variation in the winter species richness or density of species that do not regularly use supplementary feeders (model averaged partial r^2^<0.01; Table 3). In breeding assemblages, the richness, and density, of species that regularly use feeders was positively associated with the presence of supplementary feeding stations (model averaged partial r^2^ = 0.07), and canopy cover explained comparable variation in the richness of these species (model averaged partial r^2^ = 0.07). We found no evidence that the presence of supplementary feeding stations influenced the breeding richness, and density, of the species that do not regularly use feeders (model averaged partial r^2^<0.01), and the richness, and density, of these species were respectively influenced most by the proportion of green space and mean tree height (respective model averaged partial r^2^ = 0.08 and 0.1; Table 3).

**Table 3:** The results of the generalised linear model analyses of avian assemblages categorised based on whether the species is a supplementary feeding species (suppl. feeders) or non-supplementary feeding species (non-suppl. feeders), as a function of metrics of habitat quality and the occurrence of supplementary feeding stations.

		Canopy cover		Mean tree height		Supplementary feeding stations		Green space	
Avian response variable	Model average R^2^	Partial R^2^	Mean ± s.e.	Partial R^2^	Mean ± s.e.	Partial R^2^	Mean ± s.e.	Partial R^2^	Mean ± s.e.
Winter richness (suppl. feeders)	0.184	0.070	0.039±0.015	<0.001	−0.003±0.018	0.030	0.920±0.540	0.021	0.016±0.012
Winter richness (non-suppl. feeders)	0.245	0.021	0.006±0.006	0.027	0.037±0.027	0.001	−0.026±0.061	0.077	0.011±0.003
Breeding season richness (suppl. feeders)	0.203	0.071	0.039±0.012	<0.001	−0.003±0.020	0.074	1.293±0.458	0.017	0.014±0.012
Breeding season richness (non-suppl. feeders)	0.246	0.021	0.006±0.005	0.027	0.038±0.027	0.002	−0.044±0.084	0.078	0.011±0.003
Winter density (suppl. feeders)	0.127	0.022	0.111±0.100	0.001	−0.064±0.181	0.041	6.844±3.171	0.031	0.110±0.080
Winter density (non-suppl. feeders)	0.173	0.001	0.003±0.010	0.093	0.398±0.107	0.004	−0.386±0.626	0.020	0.027±0.020
Breeding season density (suppl. feeders)	0.158	0.024	0.124±0.094	0.001	−0.081±0.181	0.073	9.457±2.865	0.023	0.094±0.076
Breeding season density (non-suppl. feeders)	0.172	0.001	0.003±0.010	0.095	0.401±0.107	0.003	−0.313±0.546	0.019	0.026±0.020

An information theoretic approach to model selection was adopted and data reported are model averaged values; standard errors are unconditional.

## Discussion

Grey squirrels predate birds’ nests [14]. It has been widely suggested that this predation can alter the composition of avian assemblages and reduce the abundance of those species that are sensitive to nest predation [15]. Empirical data are limited to rural environments with some evidence that at the 1 km × 1 km spatial scale grey squirrels may slightly reduce the population size of a small number of bird species [17]. The strongest evidence that we found for such impacts in urban assemblages was that the presence of grey squirrels reduced the slope of the relationship between canopy cover and the density and richness of species most sensitive to nest predation by grey squirrels. Such relationships were not detected amongst species that were less sensitive to nest predation. Therefore, and whilst explanatory capacity was limited, the presence of grey squirrels appears to be having some influence on the structure of urban bird assemblages. All the species in our study that are sensitive to grey squirrel nest predation occur in woodland environments, and notably measures related to the abundance or type of green space, including canopy cover, had consistently stronger impacts on the size and species richness of urban breeding bird assemblages.

Grey squirrel population size and distribution in urban areas are positively influenced by supplementary feeding stations [13], [45], at which they can outcompete numerous bird species, restricting their food intake rates by over 90%, and generating potential for interference competition [16]. Despite this we find no evidence, when taking habitat availability and type into account, that grey squirrels reduced the abundance or species richness of wintering avian assemblages in a highly urbanised region. This remains the case even when assemblage composition was restricted to those species most sensitive to food competition with grey squirrels. This may be a consequence of the high abundance of supplementary feeding stations, with approximately half of UK households feeding garden birds [19], as this will increase the probability of a bird displaced from one feeder by a grey squirrel rapidly finding an alternative food source, thus limiting the impact of competition on food intake rates. The apparent lack of adverse impacts of grey squirrels on birds using supplementary feeding stations could also arise from relatively low densities of grey squirrels. Our results thus do not preclude the possibility that grey squirrels could adversely influence wintering bird populations in situations with either a lower density of supplementary feeding stations or higher squirrel densities.

We find negligible evidence that the species richness or density of bird species is influenced by supplementary feeders, which may also contribute to the lack of adverse impacts of interference competition from grey squirrels on the structure of wintering avian assemblages. In contrast, the density and species richness of breeding bird species were positively associated with the presence of feeders, supporting the current advocacy that supplementary feeding should occur in spring and summer. The explanatory capacity of the associations between feeders and breeding species richness and density were, however, rather limited, especially in comparison to previous work suggesting that supplementary feeding explains nearly half of the variation in the densities of breeding avian assemblages in Sheffield [11]. The two studies were conducted five years apart, and it is possible that changes in the nature or extent of supplementary feeding by the public, or avian responses to it, may have changed over that time period. Supplementary feeding may, for example, have become sufficiently common that the occurrence of feeders now has less of a role in regulating avian population size than was previously the case. Methodological differences, such as the use of direct observations of feeder presence (this study) in comparison to estimated densities of feeders based on socio-economic variables [11], are though perhaps more likely to contribute to the variation in results of the two studies. Although both studies indicate that breeding bird assemblages benefit from supplementary feeding the magnitude of this effect may be less marked than previously thought.

### Conclusions and management implications

We find negligible evidence that interference competition from grey squirrels at supplementary feeding stations influences the structure of urban bird assemblages during the winter, which may in part be a consequence of negligible benefits of supplementary feeders to these assemblages. During the breeding season avian densities and species richness, of those species that used feeders, responded positively to the presence of supplementary feeders. Grey squirrel occurrence reduced the densities and species richness, of those species sensitive to nest predation, by limiting the beneficial impacts of woodland cover. The positive effects of feeders and the negative effects of grey squirrels were, however, rather limited in their explanatory capacity. The amount of green space and its quality, i.e. canopy cover, exerted a stronger influence on urban bird assemblages during both the winter and breeding seasons. Conservation management activities in the focal region targeting urban bird populations should, given the current densities of grey squirrels, focus on improving the availability of high quality habitat rather than controlling grey squirrels or further increasing supplementary feeding.

## Supporting Information

Table S1 **Species classifications and attributes of their detectability functions obtained from distance sampling.**
(DOCX)

Table S2 **Assessments of how taking spatial autocorrelation into account influences full models that assess the responses of avian assemblages to grey squirrels.**
(DOCX)

Table S3 **Assessments of how taking spatial autocorrelation into account influences full models that assess the responses of avian assemblages to supplementary feeders.**
(DOCX)
