# Supplementary material for: Relative Roles of Grey Squirrels, Supplementary Feeding, and Habitat in Shaping Urban Bird Assemblages
Source: PLoS One. 2014 Oct 22;9(10):e109397. doi: 10.1371/journal.pone.0109397 (PMC4206278; doi:10.1371/journal.pone.0109397)
Supplement: Table S1 — Species classifications and attributes of their detectability functions obtained from distance sampling. (DOCX) [file pone.0109397.s001.docx]

**Table S1**. The number of detections of each bird species (water dependent species are excluded) recorded during point counts in urban Sheffield. Breeding season detections are for spring in most cases, but summer counts were used for later arriving migrants (indicated with *). When the number of detections was insufficient to construct a detectability function surrogate species were used following references [36 & 39]. Covariates that improved the fit of detectability functions are also listed. Species were counted as using supplementary feeders if they were recorded using them in at least 75% of gardens in recent years in the BTO’s Garden Bird Feeding Survey (2012) (http://www.bto.org/volunteer-surveys/gbfs/results) or, for those species not recorded in this survey, were listed as using feeders by reference [11]; the resultant classification is identical to reference [11] except that coal tit and long-tailed tit are also classified as using supplementary feeders. Species that used supplementary food were classified as sensitive to food competition from grey squirrels if they were unaggressive species that consumed most of their supplementary food from raised or hanging feeding stations, rather than feeding on the ground, as these are the types typically used by grey squirrels. This follows the results from a food competition experiment [16] which demonstrates that all small bodied passerines, with the exception of the robin, that used hanging feeders were susceptible to interference competition from grey squirrels. Predation is the main cause of nest failure and species were considered most sensitive to nest predation if their daily nest failure rates were greater than 1% nest/day as calculated from the BTO’s nest record card scheme [40]. For pheasant, data from the Game and Wildlife Conservation Trust [41] is used; as BTO do not have data for this species. Using this methodology cavity nesters and aggressive large bodied species were typically classified as least sensitive to predation whilst other species were classified as being most sensitive to predation.

| species | # winter detections | # breeding season detections | surrogate species | covariates  (other than distance) | uses supplementary feeders | sensitivity to food competition | sensitivity to nest predation |
| --- | --- | --- | --- | --- | --- | --- | --- |
| Blackbird *Turdus merula* | 139 | 170 | - | - | yes | least | most |
| Blackcap *Sylvia atricapilla* | 0 | 16* | wren | - | no | - | most |
| Blue tit *Cyanistes caeruleus* | 181 | 127 | - | - | yes | most | least |
| Bullfinch *Pyrrhula pyrrhula* | 3 | 1 | chaffinch | - | no | least | most |
| Carrion crow *Corvus corone* | 102 | 50 | - | urban form | yes | least | least |
| Chaffinch *Fringilla coelebs* | 47 | 36 | - | - | yes | most | most |
| Chiffchaff *Phylloscopus collybita* | 0 | 22 | - | detection type | no | - | most |
| Coal tit *Periparus ater* | 6 | 5 | blue tit | - | yes | most | least |
| Collared dove *Streptopelia decaocto* | 48 | 33 | - | - | yes | least | most |
| Common whitethroat *Sylvia communis* | 0 | 12* | wren | - | no | - | most |
| Dunnock *Prunella modularis* | 54 | 40 | - | urban form | yes | least | most |
| Feral pigeon *Columba livia* | 212 | 73 | - | - | yes | least | least |
| Fieldfare *Turdus pilaris* | 12 | 0 | blackbird | - | no | least | - |
| Garden warbler *Sylvia borin* | 0 | 4* | wren | - | no | - | most |
| Goldcrest *Regulus regulus* | 2 | 1 | blue tit | - | no | least | most |
| Goldfinch *Carduelis carduelis* | 119 | 74 | - | - | yes | most | most |
| Great spotted woodpecker *Dendrocopos major* | 3 | 1 | blackbird | - | no | least | least |
| Great tit *Parus major* | 66 | 49 | - | - | yes | most | least |
| Green woodpecker *Picus viridis* | 1 | 0 | blackbird | - | no | least | - |
| Greenfinch *Chloris chloris* | 74 | 41 | - | - | yes | most | most |
| House martin *Delichon urbicum* | 0 | 31* | swift | - | no | - | least |
| House sparrow *Passer domesticus* | 229 | 223 | - | detection type | yes | most | least |
| Jackdaw *Corvus monedula* | 47 | 22 | - | - | no | least | least |
| Jay *Garrulus glandarius* | 9 | 0 | - | - | no | least | - |
| Kestrel *Falco tinnunculus* | 3 | 1 | wood pigeon | - | no | least | least |
| Long-tailed tit *Aegithalos caudatus* | 91 | 27 | - | - | yes | most | most |
| Magpie *Pica pica* | 213 | 126 | - | - | yes | least | least |
| Mistle thrush *Turdus viscivorus* | 5 | 1 | blackbird | - | no | least | most |
| Nuthatch *Sitta europaea* | 3 | 0 | great tit | - | no | least | - |
| Pheasant *Phasianus colchicus* | 0 | 8 | woodpigeon | - | no | - | most |
| Pied wagtail *Motacilla alba* | 4 | 1 | starling | - | no | least | least |
| Redwing *Turdus iliacus* | 175 | 0 | - | - | no | least | - |
| Robin *Erithacus rubecula* | 154 | 89 | - | detection type | yes | least | most |
| Rook *Corvus frugilegus* | 49 | 3 | - | - | no | least | least |
| Skylark *Alauda arvensis* | 0 | 1 | house sparrow | - | no | - | least |
| Song thrush *Turdus philomelos* | 7 | 7 | - | - | no | least | most |
| Sparrowhawk *Accipiter nisus* | 1 | 1 | wood pigeon | - | no | least | least |
| Starling *Sturnus vulgaris* | 169 | 60 | - | - | yes | least | least |
| Swallow *Hirundo rustica* | 0 | 4* | starling | - | no | - | least |
| Swift *Apus apus* | 0 | 138* | - | - | no | - | least |
| Treecreeper *Certhia familiaris* | 3 | 2 | blue tit | - | no | least | most |
| Willow warbler *Phylloscopus trochilus* | 0 | 1* | chiffchaff | - | no | - | most |
| Wood pigeon *Columba palumbus* | 281 | 163 | - | - | yes | least | most |
| Wren *Troglodytes troglodytes* | 40 | 92 | - | detection type | no | least | most |
