# Supplementary material for: Relative Roles of Grey Squirrels, Supplementary Feeding, and Habitat in Shaping Urban Bird Assemblages
Source: PLoS One. 2014 Oct 22;9(10):e109397. doi: 10.1371/journal.pone.0109397 (PMC4206278; doi:10.1371/journal.pone.0109397)
Supplement: Table S2 — Assessments of how taking spatial autocorrelation into account influences full models that assess the responses of avian assemblages to grey squirrels. (DOCX) [file pone.0109397.s002.docx]

Table S2. Comparison of full models assessing responses of avian assemblages to grey squirrels, with and without taking spatial autocorrelation into account, for which the response variables exhibit statistically significant Moran’s I values (*P* < 0.05; Moran’s I <0.047 in all cases). Taking spatial autocorrelation into account (using an autocovariate model constructed in the spdep package; R v. 2.15.1, 2012) has little influence on parameter estimates (mean ± s.e.) and explanatory capacity (in parentheses). Wintering assemblages are classified according to species sensitivity to interference competition, and breeding assemblages are classified according to species sensitivity to nest predation.

| Model | Model R^2^ | Canopy cover | Mean tree height | Green space | Supple-mentary feeding stations | Squirrel occurrence | Canopy cover* squirrel occur. | Mean tree height* squirrel occur. | Green space* squirrel occur. | Suppl. feeding stations* squirrel occur. |
| --- | --- | --- | --- | --- | --- | --- | --- | --- | --- | --- |
| Winter spp. rich most sensitive species non-spatial model | 0.343 | 0.030 ± 0.008 (0.133) | -0.062 ± 0.035 (0.023) | 0.013 ± 0.005 (0.038) | -0.317 ± 0.243 (0.036) | 2.907 ± 1.460 (0.053) | 0.036 ± 0.022 (0.013) | -0.052 ± 0.104 (0.001) | -0.032 ± 0.021 (0.011) | -1.493 ± 0.807 (0.017) |
| Winter spp. rich most sensitive species spatial model | 0.344 | 0.030 ± 0.008 (0.131) | -0.062 ± 0.035 (0.023) | 0.013 ± 0.005 (0.038) | -0.312 ± 0.245 (0.036) | 2.1897 ± 1.466 (0.054) | 0.036 ± 0.022 (0.014) | -0.049 ± 0.105 (0.001) | -0.032 ± 0.021 (0.012) | -1.484 ± 0.810 (0.017) |
| Winter spp. rich least sensitive species non-spatial model | 0.224 | 0.008 ± 0.014 (0.004) | 0.115 ± 0.066 (0.019) | 0.023 ± 0.009 (0.038) | 1.242 ± 0.453 (0.045) | 5.776 ± 2.718 (0.028) | 0.013 ± 0.041 (0.001) | -0.178 ± 0.193 (0.017) | -0.036 ± 0.039 (0.005) | -1.462 ± 1.502 (0.006) |
| Winter spp. rich least sensitive species spatial model | 0.232 | 0.011 ± 0.014 (0.005) | 0.098 ± 0.067 (0.014) | 0.020 ± 0.010 (0.025) | 1.177 ± 0.456 (0.040) | 5.269 ± 2.751 (0.023) | 0.008 ± 0.041 (0.001) | -0.161 ± 0.193 (0.013) | -0.030 ± 0.039 (0.004) | -1.280 ± 1.508 (0.005) |
| Breeding spp. rich most sensitive species non-spatial model | 0.363 | 0.057 ± 0.013 (0.097) | -0.071 ± 0.065 (0.010) | 0.024 ± 0.008 (0.049) | -0.397 ± 0.398 (0.007) | 2.846 ± 1.993 (0.068) | -0.059 ± 0.026 (0.025) | -0.014 ± 0.109 (<0.001) | 0.002 ± 0.022 (<0.001) | 0.895 ± 0.898 (0.005) |
| Breeding spp. rich most sensitive species spatial model | 0.381 | 0.057 ± 0.013 (0.097) | -0.062 ± 0.065 (0.007) | 0.018 ± 0.009 (0.026) | -0.328 ± 0.395 (0.005) | 2.634 ± 1.974 (0.062) | -0.060 ± 0.026 (0.025) | -0.012 ± 0.108 (<0.001) | 0.005 ± 0.022 (<0.001) | 0.803 ± 0889 (0.003) |
| Breeding density most sensitive species non-spatial model | 0.346 | 0.146 ± 0.035 (0.087) | -0.144 ± 0.181 (0.004) | 0.042 ± 0.023 (0.021) | 0.654 ± 1.105 (0.009) | 10.330 ± 5.530 (0.076) | -0.118 ± 0.072 (0.014) | 0.178 ± 0.303 (0.002) | -0.093 ± 0.061 (0.012) | 2.010 ± 2.492 (0.004) |
| Breeding density most sensitive species spatial model | 0.382 | 0.145 ± 0.035 (0.085) | -0.116 ± 0.177 (0.002) | 0.022 ± 0.024 (0.009) | 0.744 ± 1.078 (0.008) | 9.051 ± 5.413 (0.059) | -0.117 ± 0.070 (0.013) | 0.187 ± 0.296 (0.002) | -0.082 ± 0.060 (0.009) | 1.722 ± 2.432 (0.002) |
