# Supplementary material for: Relative Roles of Grey Squirrels, Supplementary Feeding, and Habitat in Shaping Urban Bird Assemblages
Source: PLoS One. 2014 Oct 22;9(10):e109397. doi: 10.1371/journal.pone.0109397 (PMC4206278; doi:10.1371/journal.pone.0109397)
Supplement: Table S3 — Assessments of how taking spatial autocorrelation into account influences full models that assess the responses of avian assemblages to supplementary feeders. (DOCX) [file pone.0109397.s003.docx]

**Table S3**. Comparison of full models assessing responses of avian assemblages to the presence of supplementary feeders, with and without taking spatial autocorrelation into account, for which the response variables exhibit statistically significant Moran’s I values (*P* < 0.05; Moran’s I <0.045 in all cases). Taking spatial autocorrelation into account (using an autocovariate model constructed in the spdep package; R v. 2.15.1, 2012) has little influence on parameter estimates (mean ± s.e.) and explanatory capacity (in parentheses).

| Model | Model R^2^ | Canopy cover | Mean tree height | Green space | Supplementary feeding stations |
| --- | --- | --- | --- | --- | --- |
| Breeding spp. rich of supplementary-feeding species: non-spatial model | 0.249 | 0.030 ± 0.011 (0.045) | -0.049 ± 0.052 (0.005) | 0.020 ± 0.008 (0.038) | 1.182 ± 0.349 (0.064) |
| Breeding spp. rich of supplementary-feeding species: spatial model | 0.283 | 0.032 ± 0.010 (0.049) | -0.044 ± 0.051 (0.004) | 0.013 ± 0.008 (0.013) | 1.139 ± 0.342 (0.059) |
| Breeding density of supplementary-feeding species: non-spatial model | 0.211 | 0.076 ± 0.050 (0.014) | -0.302 ± 0.243 (0.009) | 0.087 ± 0.036 (0.035) | 6.647 ± 1.640 (0.096) |
| Breeding density of supplementary-feeding species: spatial model | 0.247 | 0.089 ± 0.049 (0.018) | -0.310 ± 0.239 (0.010) | 0.051 ± 0.038 (0.010) | 6.221 ± 1.617 (0.083) |
